# Identification of FRA-1 as a novel player in pancreatic cancer in cooperation with a MUC1: ERK signaling axis

**DOI:** 10.18632/oncotarget.9557

**Published:** 2016-05-23

**Authors:** Ryan L. Hanson, Roger B. Brown, Maria M. Steele, Paul M. Grandgenett, James A. Grunkemeyer, Michael A. Hollingsworth

**Affiliations:** ^1^ Eppley Institute for Research in Cancer and Allied Diseases, University of Nebraska Medical Center, Omaha, NE 68198, USA; ^2^ University of New Mexico, Albuquerque, NM 87131, USA

**Keywords:** FRA-1, MUC1, ERK, pancreatic cancer, invasion

## Abstract

The MUC1 glycoprotein is overexpressed and aberrantly glycosylated in >90% of pancreatic ductal adenocarcinoma cases and impacts tumor progression by initiating downstream signaling through phosphorylation of its cytoplasmic tail. Previous studies have demonstrated that MUC1 alters expression of known targets of activator protein 1 (AP-1); however, no studies have evaluated the precise impact of MUC1 signaling on the activity and formation of AP-1. Given the known role of these proteins in modulating migration, invasion, and tumor progression, we explored the effects of MUC1 on AP-1 dimer formation and function. We determined that MUC1 increased the protein levels of c-Jun, the major component of AP-1, and promoted dimerization of c-Jun with the Fos-protein FRA-1. We demonstrate that FRA-1 acts as a potent mediator of migration and invasion in a manner that is modulated by signals through MUC1, which acts as a dominant regulator of specific AP-1 and FRA-1 target genes. Our results provide the first *in vivo* evidence of a FRA-1 mediated expression profile that impacts pancreatic tumor growth properties. In summary, we show that MUC1 enhancement of ERK activation influences FRA-1 activity to modulate tumor migration, invasion and metastasis in a subset of pancreatic cancer cases.

## INTRODUCTION

Pancreatic ductal adenocarcinoma (PDAC) is a prominent cause of cancer related deaths worldwide. Despite recent advances in therapeutic treatment, the prognosis of patients remains relatively unchanged, with a median survival of about 6 months and a 5-year survival of only 6% [1]. Several factors contribute to the poor outcome of pancreatic cancer, including difficulties in early diagnosis and the propensity of the cancer to metastasize to distant sites early in progression [1, 2]. As such, there is a vital need for improved understanding of the mechanisms by which pancreatic cancer cells disseminate throughout the body and potential ways to specifically target these metastatic cells. MUC1, a member of the mucin family of glycoproteins that is commonly overexpressed and aberrantly glycosylated in pancreatic cancer [3], is known to modulate the invasive and metastatic potential of cancer cells. MUC1 acts by influencing the balance of adhesive and anti-adhesive properties, and by engaging in morphogenetic signaling that modifies gene expression in response to structural and microenvironmental conditions at the cell surface [4]. MUC1 exists at the cell surface as a heterodimer comprised of a large N-terminal extracellular mucin domain that is non-covalently associated with a C-terminal domain containing a short extracellular domain, transmembrane region, and a cytoplasmic tail [5]. The cytoplasmic tail is differentially phosphorylated by different receptor tyrosine kinases and serine and threonine kinases in response to cytokine stimulation, physical interactions with counter-receptors, or other factors, and acts as a relay of signals from the cell surface to the nucleus [4–6]. In cancer, MUC1 potentiates oncogenic signaling through downstream effectors [6] and acts as a transcriptional co-regulator in conjunction with transcription factors such as p53, β-catenin, and c-Jun [6–8]. This wide range of interaction partners allows MUC1 to act as a signaling hub, integrating signals from cytokine receptor status, cellular structure, and other microenviromental conditions to alter cellular behavior, including proliferation, survival, migration, and invasion [9–11].

One critical regulatory complex impacted by MUC1 is activator protein 1 (AP-1), a transcription factor comprised of Jun and Fos, which were among the first oncogenic proteins discovered [12, 13]. The Jun family of proteins includes c-Jun, JunB, JunD, and the Fos family consists of c-Fos, FosB, FRA-1, and FRA-2 [14]. Jun and Fos proteins influence cellular behavior (and transformation) in different ways, and the function of these proteins is in part dependent on the formation of specific dimers. Jun proteins can homodimerize or form Jun:Fos heterodimers. AP-1 dimers bind to TPA response elements (TRE) within DNA to regulate transcription, though the DNA elements bound depend in part on the composition of the dimer [14, 15]. The AP-1 regulated targets, matrix metalloprotease 1 (MMP1) and connective tissue growth factor (CTGF), contain known promoter sites that are co-regulated by MUC1 [7, 16], and we have previously shown that MUC1 over-expression decreases the apparent binding of c-Jun to a CTGF promoter element [16]. However, the effects of MUC1 expression on other targets of AP-1 have not been well characterized, and the mechanism by which MUC1 displaces AP-1 from specific promoters remains unknown. Given that different AP-1 dimers bind unique promoter elements, we hypothesized that MUC1 may act to integrate morphogenetic and oncogenic signaling events by altering the composition of AP-1 dimers, which in turn regulates expression of genes associated with migration and invasion.

In this report, we found that MUC1 modulated AP-1 (c-Jun and FRA-1) activity and thereby affected the migratory and invasive properties of pancreatic cancer cells. Our results provide the first evidence that in concert with ERK activation, MUC1 modified the formation of AP-1 dimers to preferentially favor c-Jun:FRA-1, which in turn enhanced the migration and invasive potential of pancreatic cancer cells *in vitro*. We show that MUC1 acts as a dominant regulator of FRA-1 function at the CTGF promoter and promotes expression of other FRA-1 regulated genes involved in migration and invasion. Increased expression of FRA-1 mRNA and protein was also observed in clinical PDAC samples, and a subset of clinical samples exhibited a FRA-1 dependent EMT gene expression signature. Knockdown of FRA-1 significantly impacted tumor growth *in vivo*, further supporting the hypothesis that a novel MUC1: FRA-1 axis contributes to the aggressiveness of PDAC.

## RESULTS

### MUC1 increases levels of active c-Jun in tumor cells

**Figure 1:**
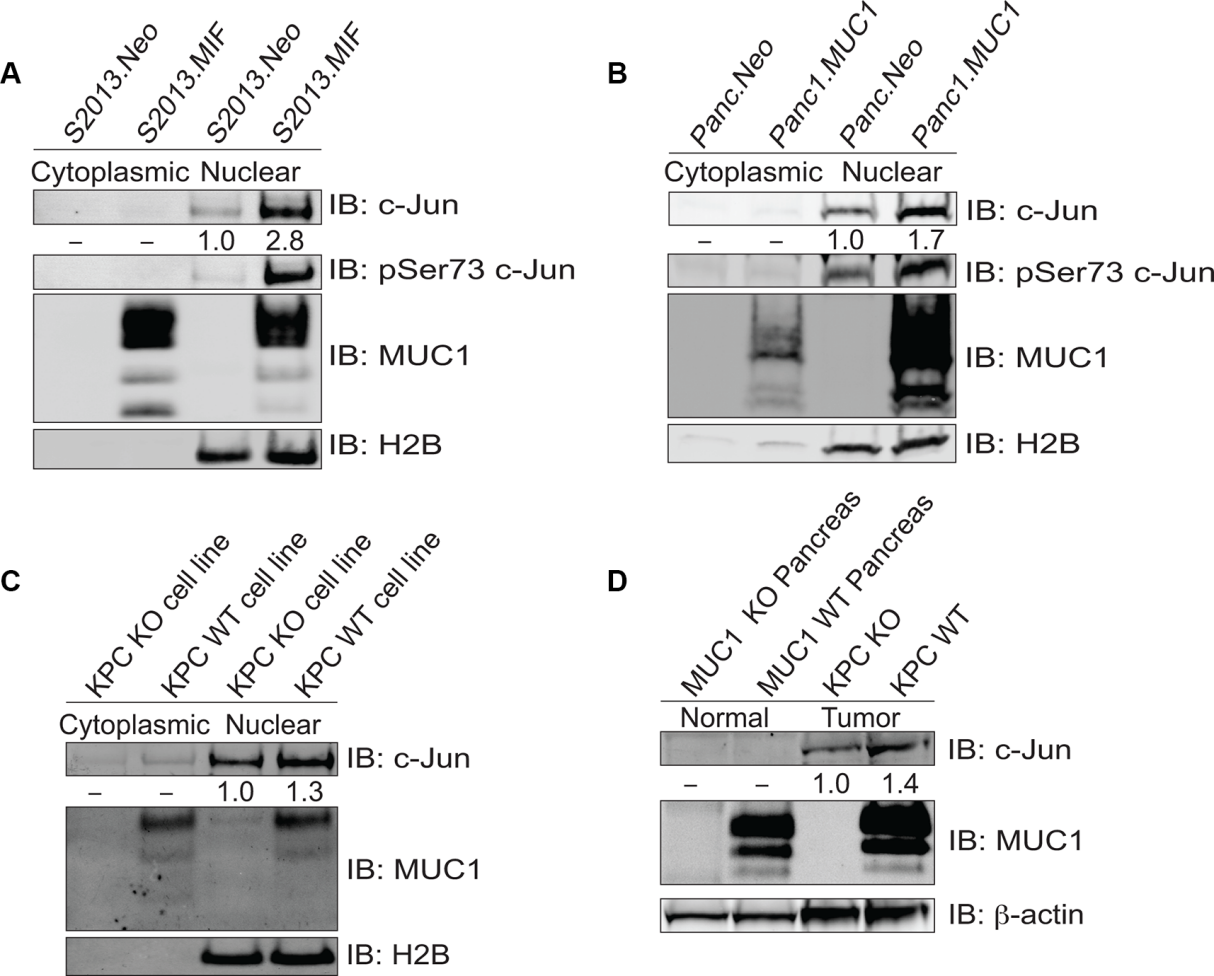
MUC1 increases expression of c-Jun protein (**A–B**) Cytoplasmic and nuclear fractions of S2013.Neo and MIF cells were western blotted for c-Jun, phosphoJun, and MUC1; H2B blotting was evaluated for normalization and purity assessment. (**C**) Cytoplasmic and nuclear fractions of cell lines established from the tumors of KPC mice that either expressed (MUC1 WT) or lacked MUC1 (MUC1 KO) were blotted for c-Jun and MUC1 expression with H2B used for normalization and purity assessment. (**D**) Whole cell lysates prepared from normal mouse pancreas and tumors derived from KPC mice (either MUC1 WT or KO) and were blotted for expression of c-Jun and MUC1 with β-actin as a loading control.

Previous studies demonstrated that MUC1 affects AP-1 regulation of target genes in pancreatic cancer cell lines [7, 16]. We initially evaluated the possibility that this was due in part to the influence of MUC1 on levels of c-Jun in two MUC1 overexpressing human pancreatic tumor cell lines, S2013.MIF and Panc1.MUC1, as compared to their low-expressing counterparts. Analysis of total c-Jun in cytoplasmic and nuclear fractions showed an increase in total c-Jun within the nucleus of MUC1 overexpressing cells (Figure 1A and 1B). MUC1 expression also resulted in increased c-Jun activation through phosphorylation at serine 73. Similarly, examination of pancreatic tumor cell lines derived from tumors of KPC mice that expressed or were null for MUC1 showed a modest increase in total c-Jun within the nucleus when MUC1 was expressed (Figure 1C). These observations were confirmed *in vivo* by analysis of normal mouse pancreas and primary pancreatic tumors derived from MUC1 expressing and MUC1-null KPC mice, which showed undetectable levels of c-Jun in normal pancreas as compared to tumor samples, and by the finding that c-Jun expression was further enhanced in tumors expressing MUC1 (Figure 1D). Unlike human tissues, normal mouse pancreas expresses high levels of MUC1.

### MUC1 promotes the formation of c-Jun:FRA-1 dimers

**Figure 2:**
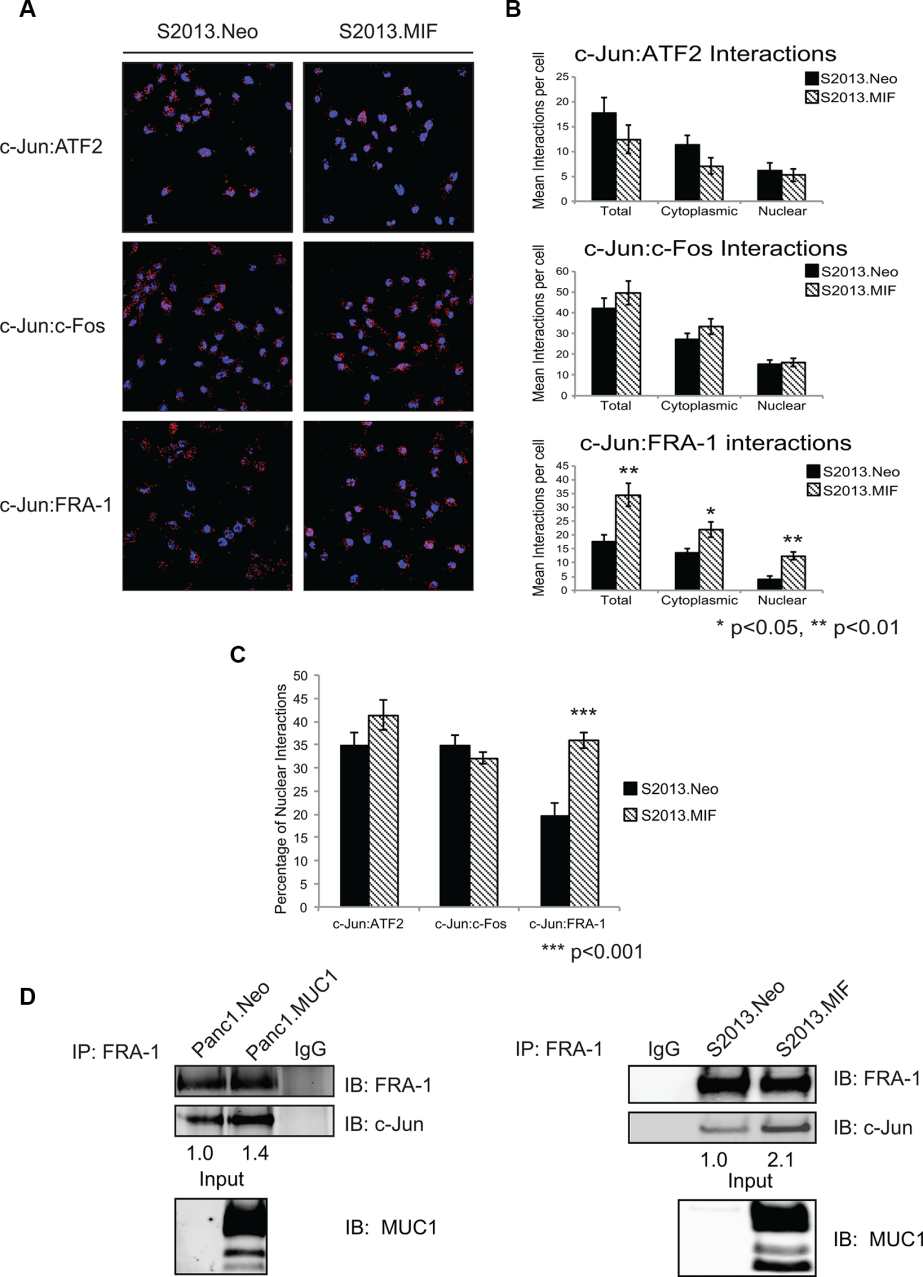
MUC1 enhances the interaction of c-Jun and FRA-1 in pancreatic cancer cells (**A**) Proximity ligation assay examining interactions of c-Jun with the proteins ATF2, c-Fos, and FRA-1 in S2013.Neo and MIF cells. Experiments were performed in four independent assays; with multiple fields were quantified for each experiment. Representative fields are shown and red dots indicate protein-protein interaction. (**B**) Quantification of interactions between c-Jun and associated partners. Quantification was performed using the Blobfinder program and results presented as the average number of interactions per cell ± SEM. Significance was assessed using two-tailed Student's *t*-test *p* < 0.05 was considered significant. (**C**) Comparison of the nuclear interactions of c-Jun and the associated proteins in S2013.Neo and MIF cells. Results represent the percentage of nuclear interactions/ total interactions ± SEM. Significance was assessed with two-tailed Student's *t*-test (**D**) FRA-1 was immunoprecipitated from Neo and MUC1 overexpressing cell lines. Immunoprecipitation studies were repeated three independent times and images are one representative experiment. The association of FRA-1 and c-Jun was then assessed by western blot analysis of co-immunoprecipitated c-Jun. Levels of c-Jun were normalized based upon amount of FRA-1 pulled down and compared between Neo and MUC1 cell lines. Western blot analysis of input confirms MUC1 expression.

We next investigated the hypothesis that MUC1-mediated increases in c-Jun levels were due to alterations in dimerization partnerships that are known to stabilize c-Jun. Previous studies have shown that MUC1 expression leads to displacement of c-Jun from promoters [7, 16]. The composition of c-Jun heterodimers is known to impact DNA binding affinity and specificity [17, 18]. We therefore evaluated AP-1 dimer composition by proximity ligation assays to assess the effect of MUC1 on interactions between c-Jun and a subset of known dimerization partners (c-Fos, FRA-1, and ATF2), which were chosen based on published roles in DNA binding, transformation, or metastatic phenotype. Representative images of PLA experiments are shown in Figure 2A. Quantification was performed using the Blobfinder program and results are presented as a representation of mean interactions per cell [19], which were further subdivided into cytoplasmic and nuclear localization (Figure 2B). MUC1 overexpression did not significantly affect nuclear interactions between c-Jun and ATF2 or c-Fos; however, c-Jun:FRA-1 interactions were significantly increased (Figure 2C). As a secondary validation that MUC1 promoted the association of c-Jun and FRA-1, we performed co-immunoprecipitation/western blotting assays to detect stable interactions between FRA-1 and c-Jun. The results showed increased amounts of c-Jun associated with FRA-1 in cells overexpressing MUC1 (Figure 2D) confirming that MUC1 promoted the association of c-Jun and FRA-1.

### MUC1, ERK, and FRA-1 regulate the migratory and invasive potential of pancreatic cancer cells

**Figure 3:**
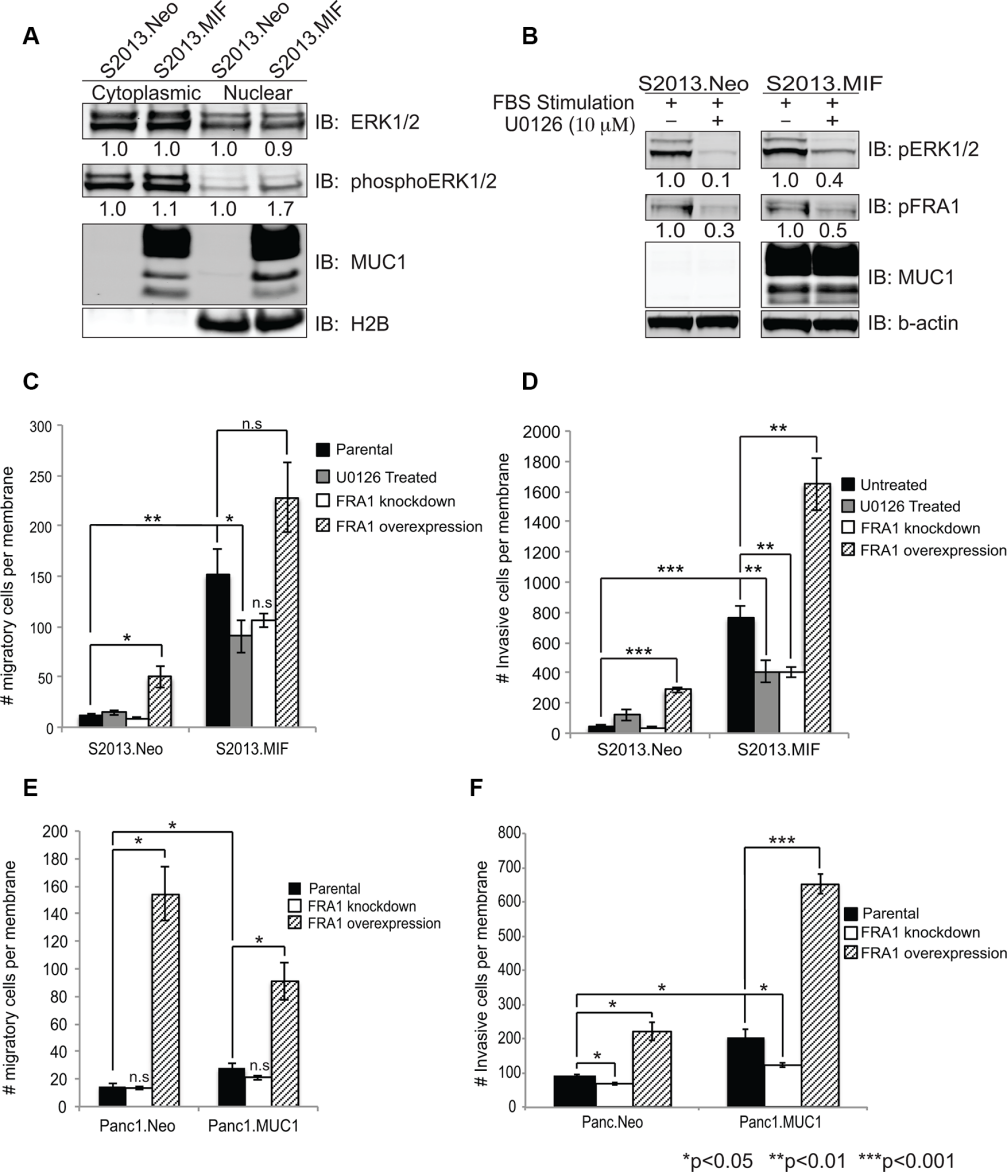
ERK activity and FRA-1 drive invasion and migration of pancreatic cancer cells (**A**) Cytoplasmic and nuclear fractions were isolated from S2013.Neo and MIF cells and western blot analysis performed for total ERK1/2, phosphoERK1/2, and MUC1. H2B was used for normalization and purity assessment. Densitometry values are shown below the Figures. (**B**) S2013.Neo and MIF cells were serum starved for 24 hours, treated with the MEK inhibitor U0126 or DMSO control for 2 hours, then stimulated with 10% FBS containing media to induce ERK activation. Western blots for phosphoERK1/2 and phosphoFRA-1 demonstrate that loss of ERK activity reduces phosphorylation of FRA-1. (**C–F**) Effects of inhibiting ERK signaling (U0126), knocking down FRA-1 mRNA, or overexpressing FRA-1 mRNA on Migration (C and E) and Invasion (D and F) in the context of low or high expression of MUC1 as assessed by Boyden chamber assays. Experiments were performed in triplicate in two independent experiments for a total of 6 data points. Plots represent number of migratory or invasive cells ± SEM. Statistical analysis was performed using two-tailed Student's *t*-test.

Upregulation of FRA-1 is commonly observed in metastatic breast cancer [20–23], where it is hypothesized that FRA-1 acts as a driver of invasion and metastatic spread of cancer cells. We sought to determine if FRA-1 played a similar role in pancreatic cancer. Given our evidence that FRA-1:c-Jun interactions increased with MUC1 overexpression, that FRA-1 can be phosphorylated via ERK, and that MUC1 is known to promote signaling through the ERK pathway [14, 24], we investigated the effects of MUC1 over-expression on ERK activation. We determined the levels of total ERK1/2 and phosphorylated ERK1/2 by western blot analysis on subcellular fractions of S2013.Neo and MIF cells. The results showed increased levels of phosphoERK2 in the nucleus of MUC1 overexpressing cells (Figure 3A). Panc1.MUC1 cells also exhibit increased ERK2 phosphorylation (Supplementary Figure 1). We confirmed that ERK activation was responsible for activation of FRA-1 by treating S2013.Neo and MIF cells with the MEK inhibitor U0126. Western blot analysis indicated that U0126 reduced phosphorylation of both ERK and FRA-1 (Figure 3B).

The results of migration and invasion assays using Boyden chamber inserts showed that inhibition of ERK signaling by U0126 treatment resulted in approximately 40% reduction in the number of migrating MUC1 expressing cells, while no effect was observed in S2013.Neo cells (Figure 3C), supporting the hypothesis that MUC1-enhanced activation of FRA-1 increased motility in pancreatic cancer cells. Similar results (increased sensitivity to loss of FRA-1 activity) were observed for the invasive potential of S2013.MIF cells (Figure 3D). We confirmed a role for FRA-1 in modulating motility by shRNA knockdown studies [confirmed by RT-PCR and western blot analysis (Supplementary Figure 2A)]. Decreased FRA-1 expression resulted in decreased migration and invasion. Similar to the effect of ERK inhibition, knockdown of FRA-1 decreased migration and invasion to the greatest degree in cells overexpressing MUC1. Conversely, overexpression of FRA-1 (Supplementary Figure 2B) greatly increased the migratory and invasive properties of S2013 cells, whether or not MUC1 was expressed, though cells overexpressing MUC1 and FRA-1 showed the highest migratory and invasive activities (Figure 3C and 3D). Similarly, overexpression of FRA-1 in Panc1.Neo or Panc1.MUC1 cells significantly increased both migration and invasion (Figure 3E and 3F), whereas knockdown decreased these properties. Notably, as for S2013 cells, these effects were higher in cells expressing higher levels of MUC1. Overexpression of FRA-1 also altered the morphology of the cells. Increased numbers of elongated cells with filipodia-like projections were observed in culture (Supplementary Figure 3A and 3B). Evaluation of these cells for proliferation revealed that at 48 hours, FRA-1 overexpression did not impact cellular growth; however, over longer time frames FRA-1 slightly enhanced proliferation as assessed by methylene blue growth assay [25] (data not shown).

### Loss of FRA-1 expression decreases tumor growth and metastases

**Figure 4:**
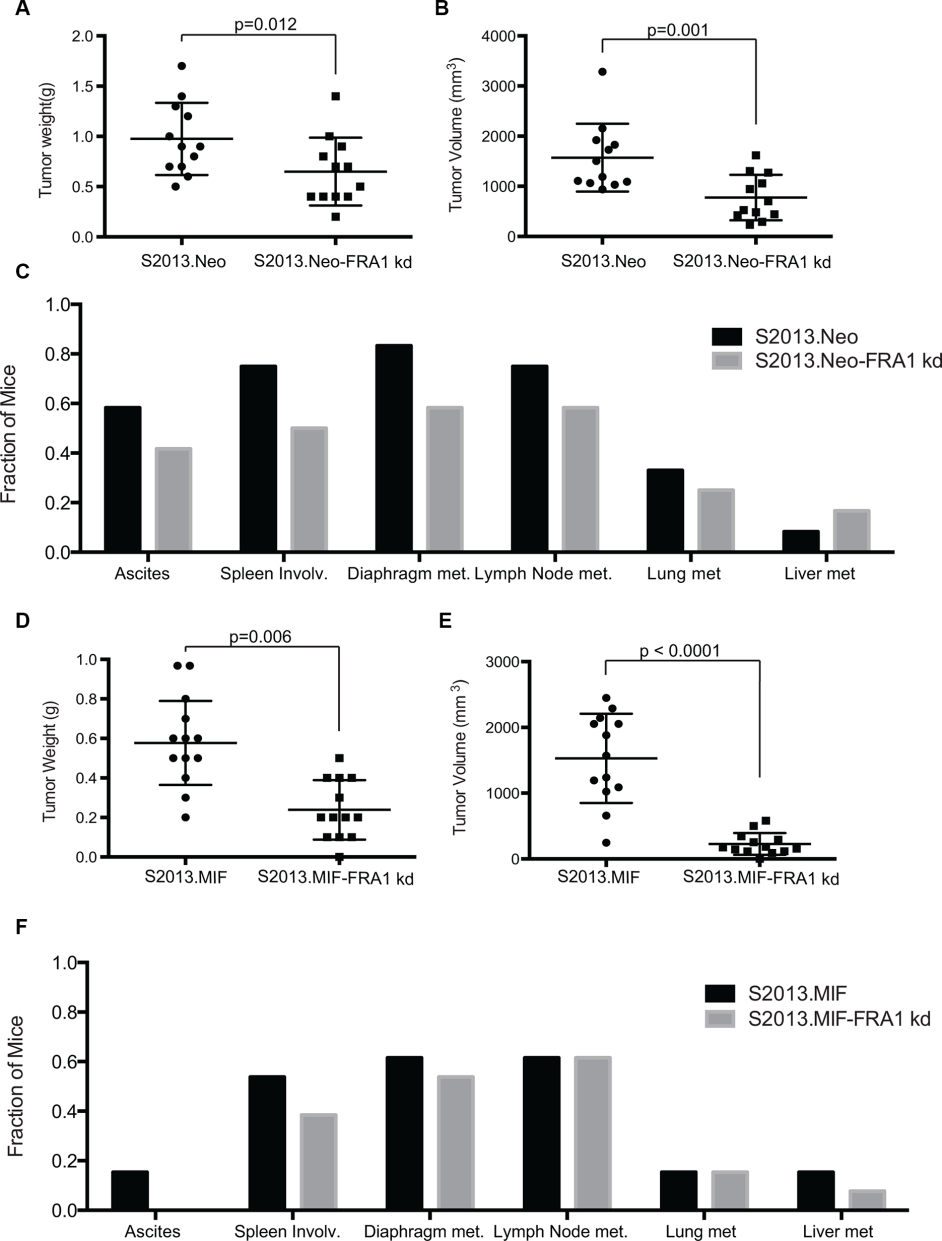
Knockdown of FRA-1 decreases tumor growth and metastasis (**A–B**) Tumor weight and tumor volume were plotted for mice injected with S2013.Neo (*n* = 12) or S2013.Neo-FRA1 kd (*n* = 12) cells. The mean was calculated ± SD. Knockdown of FRA-1 resulted in significant reduction of both weight and volume (Bonferroni adjusted *p*-values following ANOVA and Bonferroni method for multiple comparisons). (**C**) Presence of ascites or metastasis was assessed for each individual mouse and the fraction of total mice for each condition was calculated. (**D–E**) Tumor weight and volume plotted for mice injected with S2013.MIF (*n* = 13) or S2013.MIF-FRA1 kd (*n* = 13) cells. The mean was calculated ± SD (Bonferroni adjusted *p*-values following ANOVA and Bonferroni method for multiple comparisons). (**F**) Presence of ascites or metastasis was assessed and presented for each individual mouse.

To evaluate the role of FRA-1 in the development and progression of pancreatic ductal adenocarcinoma, we performed orthotopic studies evaluating tumor growth of S2013.Neo and MIF cells with or without knockdown of FRA-1 expression, using 150,000 cells injected into the pancreas of immunodeficient female nude mice. Tumors were evaluated after 30 days. Knockdown of FRA-1 expression resulted in significant reduction of primary tumor growth as assessed by both weight and volume in S2013.Neo (Figure 4A–4B). This effect was enhanced in S2013.MIF cells (Figure 4D–4E). One mouse in the S2013.MIF-FRA1 kd group failed to develop a palpable tumor, though a small tumor was detected by microscopy. Knockdown of FRA-1 also resulted in a reduction in the development of ascites.

Metastatic spread was assessed by gross analysis of tissues during necropsy and confirmed by microscopic examination of collected tissues. Knockdown of FRA-1 resulted in a 10–30% overall reduction in metastases in S2013.Neo, though some sites showed no differences, such as liver metastasis. The effects were less pronounced with high levels of MUC1 expression in S2013.MIF (Figure 4C and 4F).

### FRA-1 is upregulated in pancreatic cancer

**Figure 5:**
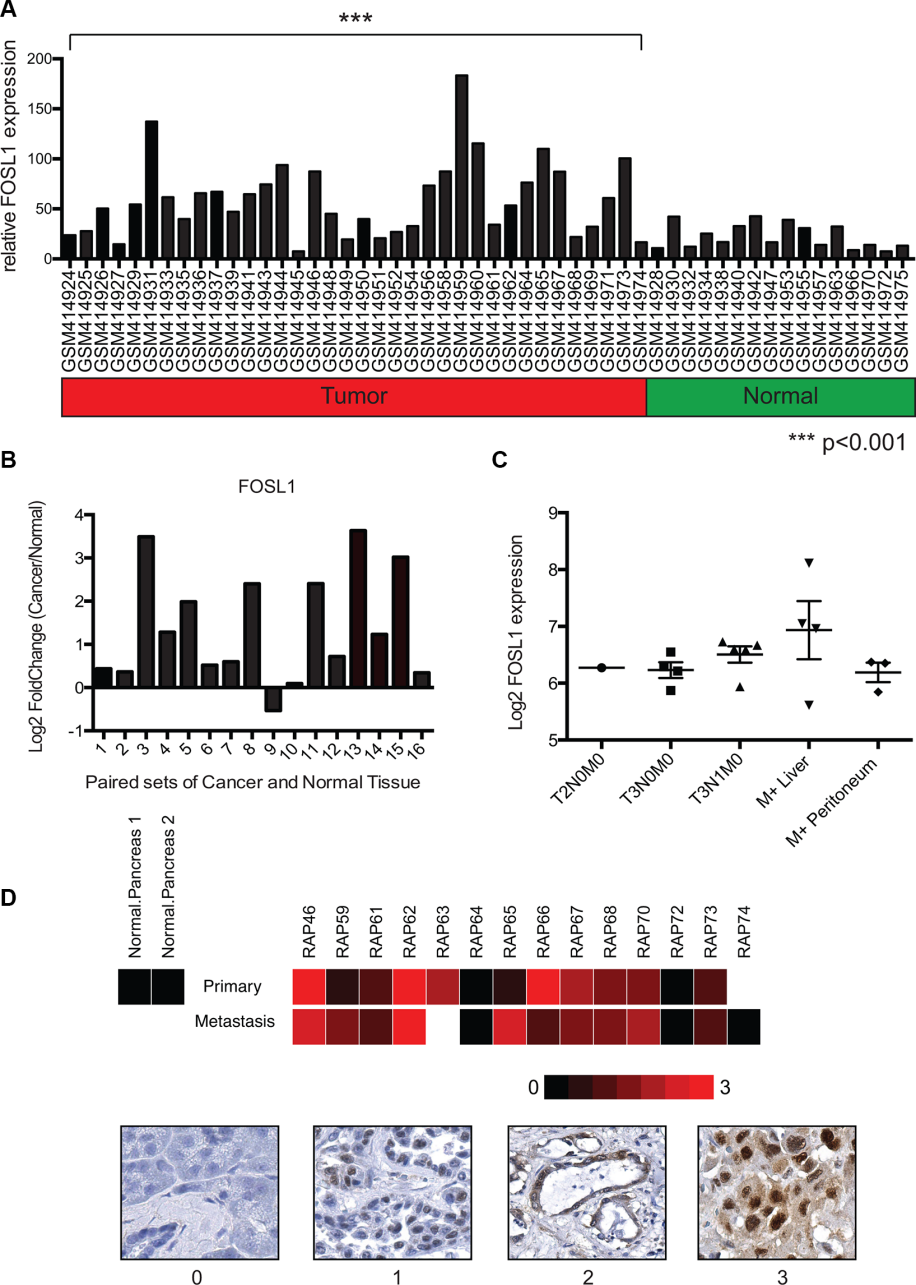
FOSL1 is overexpressed in pancreatic ductal adenocarcinoma samples (**A**) Gene expression data from the NCBI GEO dataset GSE16515 was analyzed for expression of FOSL1 (FRA-1) using microarray expression values. Expression values from tumor and normal samples are presented. Statistical comparison of the groups was performed using the Generalized Estimating Equation (GEE) to account for the paired tumor and normal samples. Analysis revealed a significant increase in FOSL1 expression in the tumor group (*p* < 0.001) as compared to normal. (**B**) To confirm tumor samples upregulated FOSL1, 16 paired tumor and normal samples were compared. The log_2_Fold Change in gene expression for tumor/normal was calculated and plotted. 15 of 16 samples show upregulation in the tumor. 8 of 16 showed an upregulation greater then 2 fold (log_2_Fold Change > 1) (**C**) Gene expression data for staged pancreatic tumor specimens was mined using the NCBI GEO dataset GSE42952. The expression of FOSL1 was plotted for each specified staging and metastatic site with absent calls ignored. (**D**) Immunohistochemistry was performed to evaluate the protein expression of FRA-1 in pancreatic cancer. A heatmap representing relative staining for FRA-1 was generated using R. Scoring was based upon intensity of stain observed only within tumor cells. Representative images for the scoring are presented below the heat map.

The role of FRA-1 in pancreatic cancer remains relatively unexplored, though it is expressed in numerous pancreatic cancer cell lines [26]. We investigated the possibility that FRA-1 contributes *in vivo* to pancreatic cancer progression by evaluating gene expression of FOSL1, which encodes FRA-1, in PDAC samples. An initial analysis included evaluation of the GEO database for microarray expression data of pancreatic ductal adenocarcinoma samples that were compared to normal pancreatic tissues. Using the data series of GSE16515, consisting of 52 samples (36 tumors and 16 normal samples), we evaluated the gene expression levels of FRA-1 [27, 28]. Analysis of relative expression levels of FOSL1 using the Generalized Estimating Equation (GEE) [29] revealed significant upregulation (*p* < 0.001) in tumors as compared to normal samples (Figure 5A). To confirm the results were not skewed by a few highly expressing tumors, we compared the 16 tumors that were matched to samples of uninvolved pancreas included in this data set. Log_2_Fold Change was utilized to compare overexpression between tumor and normal samples. We observed that 15 of 16 samples showed upregulation of FOSL1, and 8 exhibited a change of greater than 2 fold (Figure 5B).

Our *in vitro* studies suggest FRA-1 expression is important for invasive potential. To assess whether FRA-1 expression changed during the progression of pancreatic cancer, we evaluated the data series GSE42952, which includes tumor stage and some matched primary and metastatic tumors. FOSL1 expression was plotted for each tumor stage identified within the data set, ignoring absent calls (Figure 5C) [28, 30]. For metastatic sites we differentiated between the identified liver or peritoneal metastatic site. Late stage tumors showed a slightly higher trend of FOSL1 expression, particularly within liver metastases, but the low number of samples prevented us from making reliable conclusions based solely on these data. As a second evaluation, we performed immunohistochemistry using tissue microarrays of matched sets of primary and metastatic tumors derived from the UNMC Rapid Autopsy Program. FRA-1 expression was examined in primary site tumor, metastatic sites, and normal pancreas from multiple patients (Supplementary Figure 4). A heatmap was generated based upon the intensity of staining observed within tumor cells with representative images for scoring presented (Figure 5D). Most cancer cells exhibited robust nuclear staining, whereas FRA-1 was absent in normal pancreas samples; however there were no consistent trends of higher expression in metastatic samples in this limited analysis. Expression of FRA-1 also varied in different tumor samples. Thus we conclude that FRA-1 is upregulated in some but not all pancreatic cancers.

### FRA-1 overexpressing tumors exhibit a FRA-1:EMT signature

**Figure 6:**
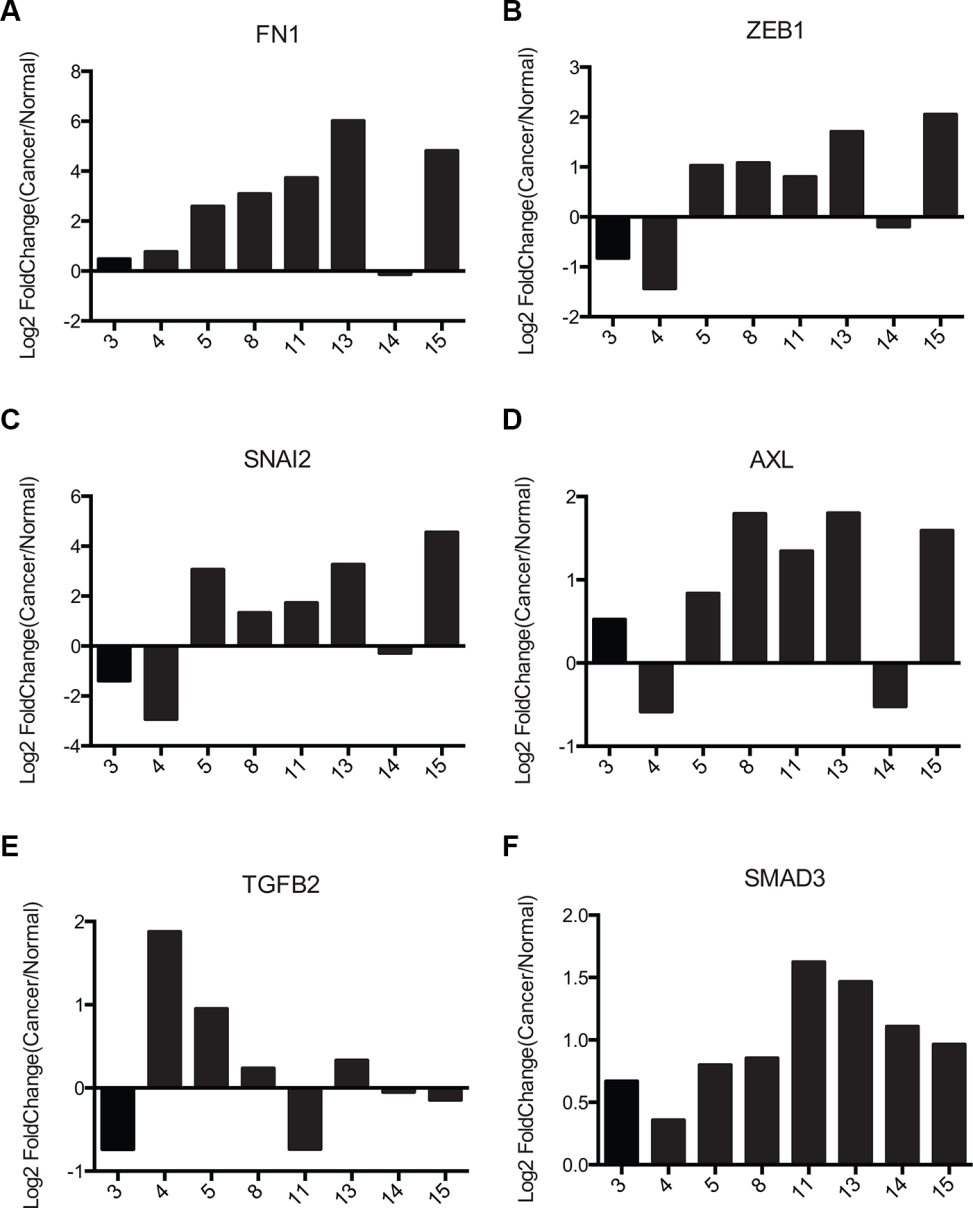
Pancreatic cancer samples exhibit similar FRA1:EMT signature as colorectal cancer cells (**A–F**) Gene expression data from the NCBI GEO dataset GSE16515 for 6 FRA1:EMT signature genes (FN1, ZEB1, SNAI2, AXL, TGFB2, SMAD3) were plotted. For genes with multiple probes the average of all probes was used. Only tumors showing > 2-fold upregulation of FRA-1 were used for this analysis.

Recently a FRA-1:EMT signature has been proposed for colorectal cancer cells overexpressing a flagged FRA-1 construct [31]. Eight genes identified as regulated by FRA-1 were found to represent part of an Epithelial to Mesenchymal Transition (EMT) associated signature: VIM, FN1, FOSL1, ZEB1, SNAI2, AXL, TGFB2, and SMAD3. We chose to examine gene expression of 6 of these genes (FN1, ZEB1, SNAI2, AXL, TGFB2, and SMAD3) in PDAC samples. The GSE16515 PDAC data set was analyzed for a similar signature. We evaluated paired samples that overexpressed FOSL1 at least two-fold, which were predicted to exhibit a FRA-1 associated phenotype [27]. Calculation of Log_2_Fold Change for each paired set of tumor and normal samples for these targets (Figure 6A–6F) showed a substantial correlation between FRA-1 expression and upregulation of these EMT signature mRNAs. 5 of the 6 targets (FN1, ZEB1, SNAI2, AXL, and SMAD3) were upregulated in at least 60% of the tumors with high FRA-1 expression; however TGFB2 showed no consistent trend. Tumor samples 5, 8, 11, 13, and 15 were most consistent with the predicted FRA-1 signature, mirroring the predicted trend 100% of the time for genes other than TGFB2. These results support the hypothesis that pancreatic cancer exhibits a FRA-1 driven EMT signature, though only within a subset of cases.

To examine whether protein expression of these genes correlated with FRA-1 in patient samples, we performed IHC for Slug (SNAI2) and ZEB1 on matched sets from our rapid autopsy program (Supplementary Figures 5 and 6). Slug was observed in most samples, though it was absent in a FRA-1 negative tumor. ZEB1 was absent in most samples, but present in a few tumors highly expressing FRA-1. A heatmap representing the IHC pattern was generated using R programming language (Supplementary Figure 7). These results suggested that even though there were effects on mRNA levels, there was not a direct correlation between expression of FRA-1 and the protein products of its target genes Slug and ZEB1 in clinical samples, demonstrating that factors other than mRNA levels influence steady state levels of these proteins.

### MUC1 regulates expression of FRA-1:EMT gene targets

**Figure 7:**
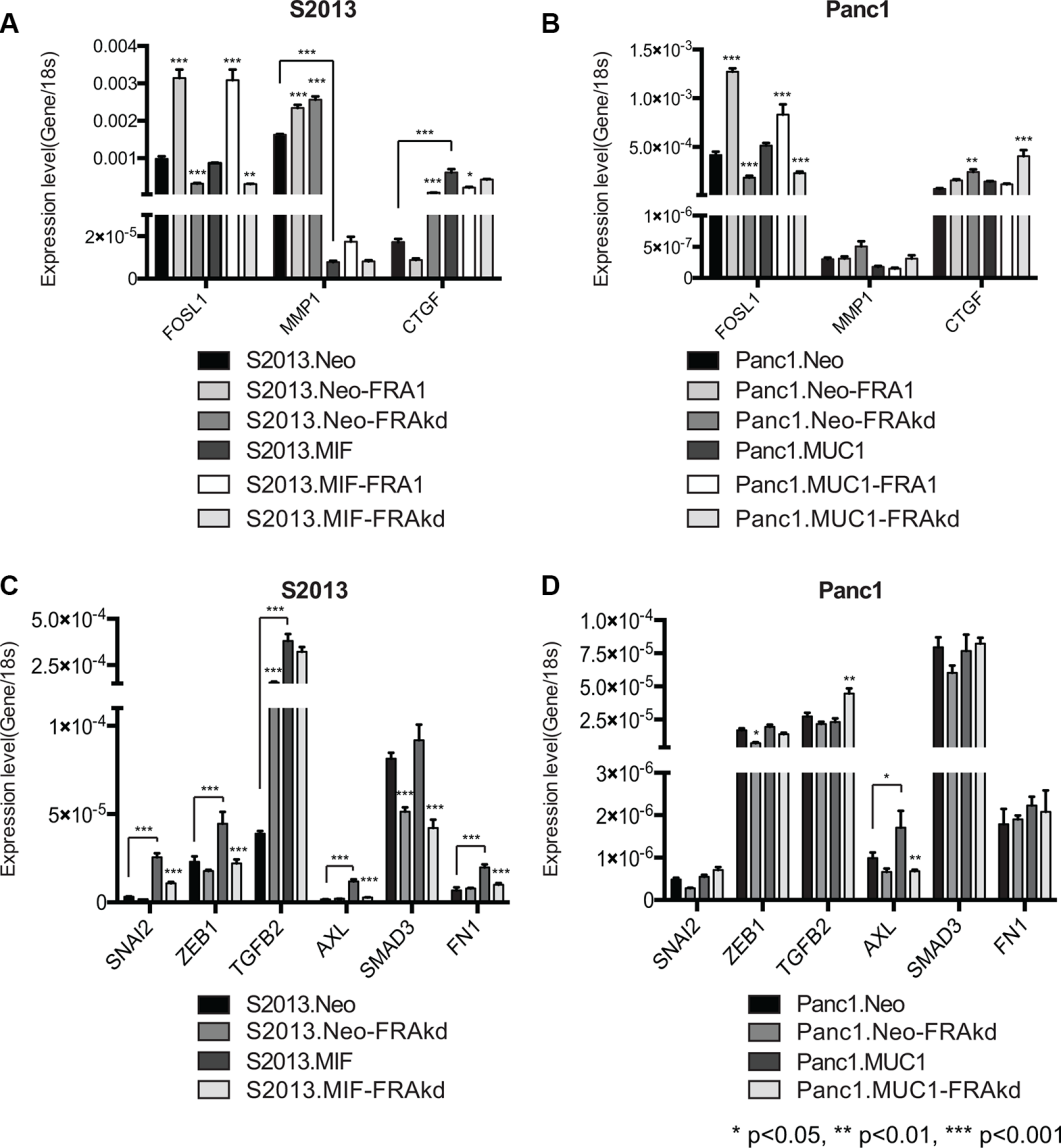
MUC1 regulates specific AP-1 and FRA-1 targets (**A–B**) RT-PCR studies were performed to examine the impact of FRA-1 on the MUC1 regulated genes MMP1 and CTGF. FOSL1 expression was measured to confirm overexpression or knockdown of FRA-1. Results were analyzed by 2-way ANOVA and *p*-values represent comparison of FRA-1 overexpression and knockdown lines to parental unless otherwise indicated by lines. MUC1 expression caused significant alterations to expression of MMP1 and CTGF. Alteration of FRA-1 expression had no impact on expression in S2013.MIF, but significantly altered expression of these genes in S2013.Neo (**C–D**) Additional RT-PCR studies were performed to examine the impact of MUC1 and FRA-1 on expression of putative FRA1:EMT genes. Results were analyzed by 2-way ANOVA for multiple comparisons and *p*-values indicate significant differences between parental and FRA-1 knockdown cell lines unless otherwise indicated by lines. In S2013 cells (C) MUC1 caused significant increases in expression of these genes. Loss of FRA-1 abrogated this effect and restored expression to levels similar to S2013.Neo cells. Similar effects were observed for the regulation of AXL in Panc1 cells (D).

As previous studies have demonstrated the capacity of MUC1 to regulate expression of MMP1 and CTGF, we sought to examine how the interplay of MUC1 and FRA-1 impacted expression of these genes[7, 32]. We performed RT-PCR analyses of our S2013.Neo, S2013.MIF, Panc1.Neo, and Panc1.MUC1 cell lines in conjunction with FRA-1 overexpression or knockdown to examine expression of MMP1 and CTGF. Expression of FOSL1 was used as a positive control to confirm overexpression or knockdown of FRA-1. Results indicated that increased MUC1 expression dramatically impacts expression of MMP1 and CTGF in S2013 cells. Overexpression or knockdown of FRA-1 did not reverse these effects, indicating a dominance of MUC1 effects at these sites, whereas loss of FRA-1 in S2013.Neo resulted in CTGF expression comparable to that observed in S2013.MIF (Figure 7A). These results were more modest in Panc1 cells and reflect a less robust impact of MUC1 on expression of these targets in this cell line (Figure 7B).

To examine whether MUC1 impacted expression of FRA-1:EMT genes, we performed additional RT-PCR studies examining expression of SNAI2, ZEB1, TGFB2, AXL, SMAD3, and FN1. Expression of MUC1 in S2013 cells caused significant upregulation of many of the FRA-1:EMT genes, including SNAI2, ZEB1, AXL, and FN1. Loss of FRA-1 caused significant reduction of these genes to levels comparable to the S2013. Neo cell line. The impact of FRA-1 knockdown in the Neo cell line was relatively modest on most FRA-1:EMT genes (Figure 7C). Once again these effects were less pronounced in the Panc1 cell lines, though AXL was impacted by MUC1 expression (Figure 7D).

## DISCUSSION

**Figure 8:**
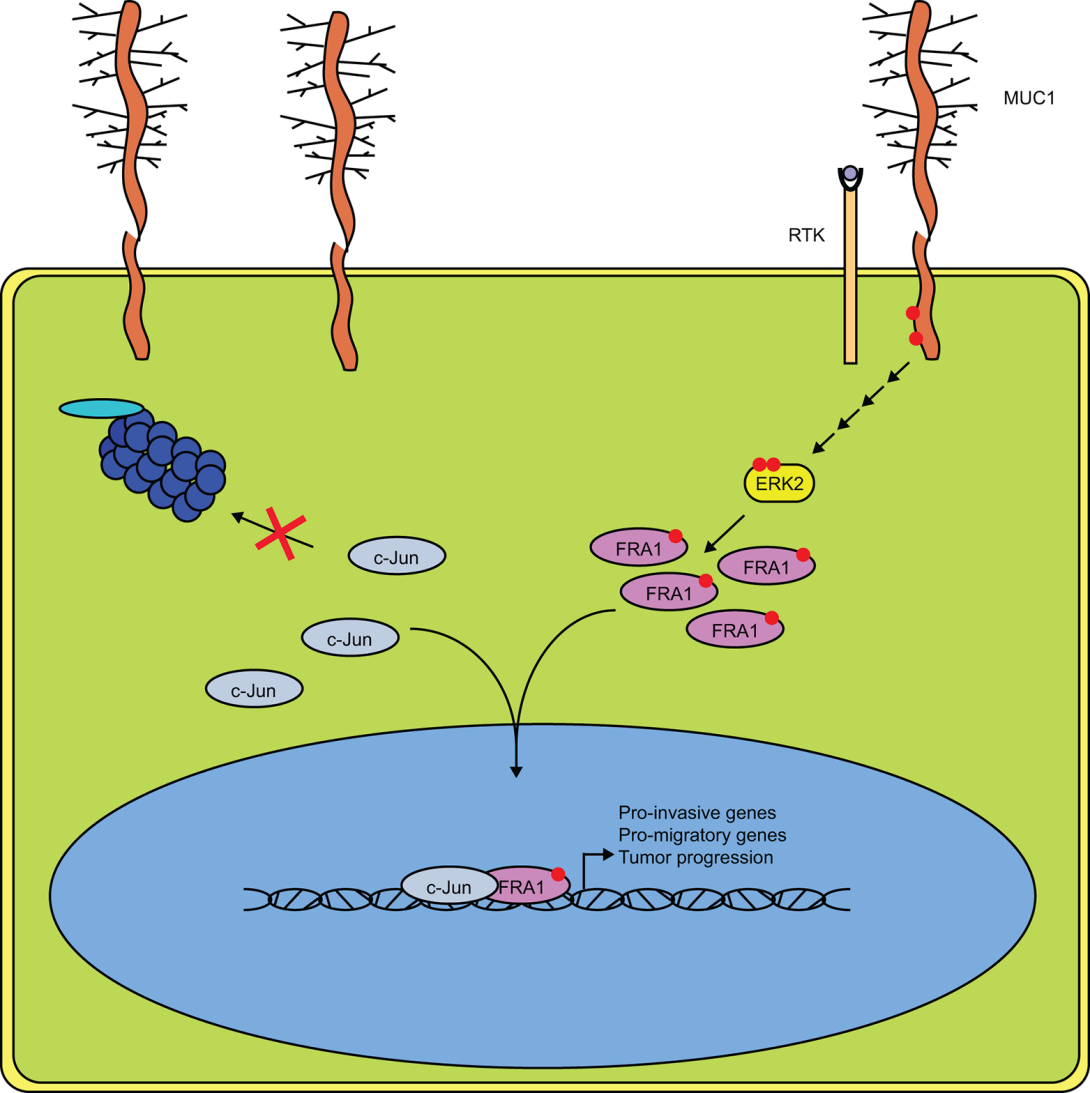
MUC1 and ERK Cooperate to Drive Association of c-Jun and FRA-1 Schematic representation of our proposed mechanism in MUC1 expressing pancreatic cancer cells. Phosphorylation of the MUC1 cytoplasmic tail drives downstream activation of ERK, likely involving the association of MUC1 with receptor tyrosine kinases. Increased ERK activity results in phosphorylation of FRA-1 and promotes dimerization with c-Jun. The transcriptional complex is then stabilized and allows for expression of genes involved in migration, invasion, and overall tumor progression.

That MUC1 affects gene expression is well established [33–36]; however, the mechanism by which MUC1 regulates transcription and affects tumor progression is not fully understood. MUC1.CT is known to interact with a wide range of transcription factors including p53, β-catenin, c-Jun, and others [7, 8, 16]. Previous studies have shown that MUC1 displaces c-Jun from promoters of known target genes; the data presented here demonstrates this effect is not the result of decreasing the levels of c-Jun within the cell. Rather, our results indicate that high levels of MUC1 alter the AP-1 transcriptome in part by increasing steady state levels of c-Jun protein. We went on to demonstrate that this stabilization of c-Jun results in enhanced association with FRA-1 in cells expressing high levels of MUC1, suggesting that MUC1 alters the stoichiometry of AP1 protein complexes, which in turn modifies transcriptional activity (Figure 8).

We found that MUC1 acts as a dominant regulator of FRA-1 activity that in turn modulates expression of CTGF and MMP1. Additionally, MUC1 upregulated expression of several genes associated with FRA-1 mediated EMT. Steady state mRNA levels for these genes were reduced upon FRA-1 knockdown. Together, these results suggest that MUC1 serves as a co-activator for FRA-1 at many FRA1:EMT sites, whereas it may function as a de-repressor of FRA-1 at the CTGF and MMP1 sites. These results were less apparent in Panc1 cells, which may be attributed to differences in context dependent constitutive signaling between the S2013 and Panc1 cell lines. Indeed, the impact of MUC1 on the migration and invasion of Panc1 cells was more modest than the effects on S2013 cells. Thus, it is not surprising that expression of genes involved in migration and invasion are not substantially altered in the Panc1 cells.

Consistent with the findings reported here, FRA-1 has been shown to impact migration, invasion, and metastasis in a number of different cancers [14, 20, 22, 31]. In particular, the role of FRA-1 is well characterized in breast cancer, which also commonly exhibits MUC1 overexpression and consequent effects on signaling. Similar to breast cancer, FRA-1 enhances the migratory and invasive capacity of pancreatic cancer cells. The finding of a cooperative effect between MUC1 and FRA-1 that resulted in substantial increases in migration and invasion addresses in part the finding that these factors show differential effects on transcription of different genes. It is likely that one function of MUC1 is to integrate morphogenetic and oncogenic signals that arise from cell surface structural conditions, cytokine and growth factor stimulation and steady state signaling within the cell [5] to help enact programs of transcriptional response to these composite sets of stimuli from the cellular microenvironment and internal signaling apparatus. Programs of cellular activity (e.g. EMT, cell migration, cell division, other cellular functions) require differential transcriptional responses (up-regulation and down-regulation of different sets of genes), and so it is not surprising that an integrator of signaling such as MUC1 would act as both a co-activator and a co-repressor. For results examined here, inhibiting ERK activation or knocking down expression of FRA-1 produced similar effects in MUC1 expressing cells, which supports the hypothesis that MUC1 integrates ERK signaling with morphometric signals related to motility and invasion. Furthermore, we demonstrated that MUC1 enhanced steady state ERK activation in pancreatic cancer cells, further supporting the link between ERK activation and the functional activity of MUC1 and FRA-1. Previous studies have also demonstrated the capacity for MUC1 to promote ERK mediated signaling, however, these studies did not evaluate the impact on downstream transcriptional machinery [24, 37]. These results serve as the first reported evidence of cooperative signaling between MUC1 and FRA-1. This finding could have important implications not only in pancreatic cancer, but also in other cancers with aberrant expression of cell surface mucin proteins that engage in signal transduction [5].

Despite the known synergy between AP-1 and oncogenic Ras, few studies have examined the expression of AP-1 proteins in pancreatic cancer, which contains K-Ras mutations in a vast majority of cases [32, 38]. Our analysis of data from the GEO database suggested that FRA-1 is transcriptionally upregulated during the progression from normal to cancerous pancreatic tissue and FRA-1 mRNA may also be upregulated as the tumor progresses to metastasis. Immunohistochemistry supported these analyses in part, as pancreatic tumors exhibited robust nuclear staining for FRA-1 and expression was retained in liver metastases. FRA-1 staining was absent in samples of normal pancreas. A set of FRA-1 target genes identified in studies of colorectal cancer studies was confirmed here for pancreatic cancer. Recently, it has been proposed that pancreatic cancer consists of 4 distinct subtypes based on genomic analyses [39]. Interestingly, the proposed squamous subtype of pancreatic cancer exhibits high expression of FOSL1, TGFB2, SNAI2, and FN1 [39], which is consistent with the FRA1:EMT phenotype we describe here. This suggests that our proposed FRA1:EMT subset may overlap with the squamous subtype of pancreatic cancer. Immunohistochemistry analysis of clinical samples obtained at autopsy provided additional support for the hypothesis that FRA-1 was associated with expression of these genes; however, it was also apparent that the levels of expression of proteins encoded by the target genes are influenced by other factors. Future studies in which pancreatic tumors are stratified according to subtypes or other features of aggressiveness may reveal correlations. Additionally, the dependence of FRA-1 activity on ERK activation suggests that tumors (such as pancreatic cancer) bearing activating mutations with the Ras-Raf-MEK-ERK cascade are likely to exhibit FRA-1 based effects.

Our results support the hypothesis that FRA-1 contributes significantly to metastasis of pancreatic cancer, at least within a subset of cases, and also plays an important role in overall tumor progression. Reduction of FRA-1 expression by as little as 2-3 fold produced significant reductions in primary site tumor growth in an orthotopic model of pancreatic cancer. Metastases were also reduced, though not completely inhibited. Recent studies have highlighted a potential role for FRA-1 in anchorage independent growth [40]. Other studies have highlighted the importance of FRA-1 in promoting YAP driven oncogenesis [41], which is important in the progression of pancreatic cancer. These results suggest FRA-1 may be a viable target to inhibit the growth and dissemination of pancreatic cancer cells. To date no specific inhibitors to FRA-1 have been characterized, though various inhibitors such as bromodomain inhibitors impact FRA-1 expression [40, 42]. As FRA-1 exhibited a number of effects independent of MUC1, future studies focused on FRA-1 alone may provide further insight into the possibility of targeting FRA-1.

In conclusion, our work presents the first evidence that MUC1 can function by altering the composition of AP-1 protein complexes involved in transcriptional regulation. This function explains some of the effects of MUC1 on the expression of genes involved in migration and invasion, particularly those that are known targets of FRA-1. We further highlight the functional role of these changes as drivers of metastatic and invasive potential in pancreatic cancer cells. Given that 90% of pancreatic cancer patients exhibit metastatic spread at diagnosis, the mechanisms behind the early dissemination of pancreatic cancer cells need further study [1]. Whereas the mechanism identified in this manuscript identifies aspects of the biology of MUC1 in modulating transcriptional effects, our *in vivo* and *in vitro* studies suggest FRA-1 can independently contribute to effects on tumor progression. Additional study of FRA-1 in pancreatic tumor specimens is warranted, especially with respect to its potential contribution to subtypes of pancreatic cancer, as is further study of the specific and redundant functions of c-Jun heterodimers in pancreatic cancer. Future studies should also be undertaken to identify potential therapeutic targets of specific AP-1 heterodimers.

## MATERIALS AND METHODS

### Cell culture

Panc1 cells were obtained from American Type Culture Collection and S2013 cells were obtained from the originator of the line [43]. S2013.Neo and MIF were generated as previously described [8]. Panc1.MUC1 and Neo were generated from stable transfection of pSIN-ires-neo using lentiviral transfection. Panc1.MUC1-FRA1, Panc1.Neo-FRA1, S2013.Neo-FRA1, and S2013.MIF-FRA1 lines were generated by stable transfection of pLVX.puro using lentiviral transfection. Cells were selected in Dulbecco's modified Eagle's medium (DMEM) supplemented with 4 μg/ml Puromycin, 10% fetal bovine serum (FBS), and 1X HyClone penicillin/streptomycin mix (100 U/ml penicillin and 100 μg/ml). Once selection had occurred cells were maintained in 10% DMEM supplemented with HyClone pen/strep mix. Cells were maintained at 37°C in a humidified environment with 5% CO_2_.

### KPC MUC1 knockout mice and cell lines

KPC mice were bred at UNMC to carry the *PDX-1-Cre* transgene [44], the *LSL-KRAS^G12D^* knock-in mutation [45] and the *LSL-Trp53^R172H^* knock-in mutation [46]. In the context of the KPC background, mice were bred to the Muc1 knockout mouse [47] to generate KPC mice that express Mucin-1 (KPC WT) or are deficient in Mucin-1 expression (KPC KO). Cell lines were derived from primary tumors of each genotype and utilized for further analysis.

### Construct Generation

FRA-1 constructs were designed by PCR amplification of FRA-1 cDNA purchased from OpenBiosystems. Primers used were designed for placement of a HA-epitope tag at the C-terminus of FRA-1. Amplified fragments were then restriction digested and ligated into pLVX.puro vector for lentiviral transfection. FRA-1 knockdown was performed using the previously characterized shRNA TRCN0000019539 [48] or scrambled control purchased from OpenBiosystems.

### Subcellular Fractionation

Cytoplasmic and Nuclear fractions were obtained using the nuclear fractionation protocol from Abcam. Cells were grown to 80–90% confluence and lysed into Buffer A (10 mM HEPES, 1.5 mM MgCl_2_, 10 mM KCl, 0.5 mM DTT, and 0.5% NP40) and then incubated on ice for 30 minutes. Lysates were spun down at 3000 rpm for 10 minutes at 4°C to pellet nuclei. The supernatant was removed as the cytoplasmic fraction and the nuclear pellet was washed 3 times in Buffer A to remove potential contaminants. The nuclear pellet was lysed in Buffer B (5 mM HEPES, 1.5 mM MgCl_2_, 0.2 mM EDTA, 0.5 mM DTT, 26% glycerol, supplemented with 300 mM NaCl added fresh). To ensure lysis, the nuclear pellet was passed through a 25-gauge needle and the lysates incubated on ice for 15 minutes. Lysates were then spun down at 16,000 g for 15 minutes to pellet insoluble debris. The supernatant was collected as the nuclear fraction. Fraction purity was assessed by western blotting with an antibody to Histone 2B.

### Co-immunoprecipitation

Cells were grown to 80–90% confluence and lysed in co-immunoprecipitation buffer (50 mM Tris-Cl, 150 mM NaCl, 0.5% NP40, pH 8.0) in the presence of protease and phosphatase inhibitors (ThermoFisher 78440). Buffer was adjusted to 300 mM NaCl to promote extraction of nuclear proteins. Lysates were incubated on ice for 30 minutes and insoluble debris removed by centrifugation at 16,000g. 300 μl (1 mg of protein) of lysate was incubated with Protein G beads (ThermoFisher 1003D) and 3 μg FRA-1 antibody at 4°C for 2 hours to form complexes. Beads were washed 3X with Co-IP wash buffer (50 mM Tris-Cl, 150 mM NaCl, 0.1% NP40) and proteins eluted by boiling in SDS sample buffer. Experiments were performed three independent times.

### Immunoblotting

Proteins were transferred from gels to Immobilon-FL PVDF membrane using the Bio-Rad transfer system at 100V, 0.3 A, for 70 minutes. Membranes were rinsed in 1X PBS then blocked for 1 hour in a 1:1 mixture of 1X PBS and Licor Blocking buffer. Primary antibodies were incubated for 1 hour in 0.1% PBST:Licor buffer. All primary antibodies were used at a concentration of 1 μg/ml. Membranes were washed three times with 0.1% PBST. Secondary antibodies were conjugated to IrDyes (Licor) and incubated for 1 hour in the dark in 0.1% PBST/Licor buffer with 0.01% SDS to reduce background. Blots were washed three more times, rinsed with 1X PBS, and visualized using the Odyssey Imaging System. Densitometry of western blots was performed using the ImageJ software. All western blots were replicated a minimum of three times.

### Antibodies

The anti-MUC1 antibody CT2 was generously provided by Dr. Sandra J Gendler or ordered from Abcam (ab80952). Antibodies against c-Jun and phosphoSerine73 c-Jun were obtained from Abcam (ab31419, ab32447). Antibodies against phosphoFRA-1, phospho-c-Fos, ERK, and phosphoERK were purchased from Cell Signaling (#5841, #5348, #9107, and #4377 respectively). FRA-1, c-Fos, and H2B were obtained from Santa Cruz (sc-28310, sc-8047, sc-8650) and ATF2 from Novus Biologicals (H00001386-M02). β-actin was obtained from Sigma Aldrich.

### RNA Isolation and RT-PCR

Cells were grown to 80–90% confluence on 15 cm dishes, rinsed with 1X PBS and RNA isolated using the Qiagen RNeasy kit. Isolated RNA was aliquoted and stored at −80°C until use. RNA was converted to cDNA using the Verso cDNA kit (ThermoFisher) and cDNA was stored at −20°C until use. RT-PCR for each primer set was performed in triplicate and Sybr Green was used for signal detection. Fold change was calculated using the ^ΔΔ^Ct method and converted to log_2_fold change. All RT-PCR primers were ordered from PrimerBank [49, 50].

### Proximity Ligation Assay (PLA)

All PLA reagents used were from Duolink PLA kit. Cells were grown in 12-well plates on poly-lysine coated slides. Cells were fixed with 4% PFA supplemented with 120 mM sucrose. The reaction was quenched by removal of PFA and addition of 0.1 M glycine for 5 minutes followed washes with 1X PBS. Cells were permeabilized using 500 μl of 0.15% Triton-X-100 with 1% BSA in 1X PBS for 15 minutes then blocked with 1 drop of blocking solution for 30 minutes at 37°C. Blocking solution was removed and coverslips incubated with the primary antibodies in antibody diluent (1:200) overnight at 4°C. After incubation, coverslips were washed three times with PLA wash buffer A for 5 minutes. PLA secondary probes were added and incubated at 37°C for 1 hour in the dark. Coverslips were washed twice more with PLA wash buffer A and ligation reaction mix was added to the coverslips for 30 minutes at 37°C. After two more washes in buffer A the amplification-polymerase solution was added for 100 minutes at 37°C. Coverslips were washed twice in 1X PLA wash buffer B then once in 0.1X buffer B. Coverslips were mounted with Fluoromount G with DAPI. Results were visualized with confocal microscopy and the interactions were quantified using Blobfinder. All experimental groups were performed in quadruplicate and multiple independent fields per slide were used for quantification.

### Invasion and migration assays

Assays were carried out using matrigel invasion plates or control migration plates from BD Biosciences (#354480 and #354578 respectively). Cells were grown to approximately 60% confluence and serum starved 24 hours prior to assay. Treated cells received a 2-hour pretreatment of 10 μM U0126 (Sigma). Prior to plating, matrigel matrix in invasion plates was rehydrated for 2–4 hours with serum free media at 37°C, migration plates required no pre-treatment. 100,000 cells per well were plated for invasion assays and 25,000 cells per well for migration assays. Chemoattractant in the lower well was 10% FBS containing DMEM. Cells were incubated at 37°C for 24 hours, then inserts were washed and stained using the DiffQuick staining kit. Membranes were dried and mounted on slides for quantification. Each experimental group was examined in two independent experiments with each group plated in triplicate.

### Immunohistochemistry

Slides containing primary pancreatic tumor, liver metastases, or uninvolved pancreas were obtained from the UNMC Rapid Autopsy Program. Staining was performed using the Dako Envision+ kit (K4006) with a hematoxylin counter-stain. Anti-FRA-1 (sc-28310), Anti-ZEB1 (ab180905), Anti-slug (ab27568) and IgG control were used at a concentration of 10 μg/ml. Following staining, slides were mounted and imaged.

### Orthotopic mouse studies

All animal studies were performed according to IACUC standards. 150,000 tumor cells were injected orthotopically into the pancreas of immunodeficient female nude mice. S2013.Neo and MIF cell lines and their FRA-1 knockdown counterparts were utilized for the study. Groups consisted of 12–13 mice for a total of 50 mice overall. Tumors were allowed to develop for 30 days, at which time mice were sacrificed and tumors measured. Presence of ascites and metastasis was initially assessed based on gross observation during necropsy. Tissues for each metastatic site and primary tumor were formalin fixed. The UNMC tissue sciences core facility cut and stained H&E slides for each sample in the experiment. Metastases were confirmed by microscopy before final scoring.

## SUPPLEMENTARY MATERIALS


