# Supplementary material for: Identification of FRA-1 as a novel player in pancreatic cancer in cooperation with a MUC1: ERK signaling axis
Source: Oncotarget. 2016 May 23;7(26):39996–40011. doi: 10.18632/oncotarget.9557 (PMC5129987; doi:10.18632/oncotarget.9557)
Supplement: Supplementary file 1 [file oncotarget-07-39996-s001.pdf]

Identification of FRA-1 as a novel player in pancreatic cancer in cooperation with a MUC1: ERK signaling axis

Supplementary Materials

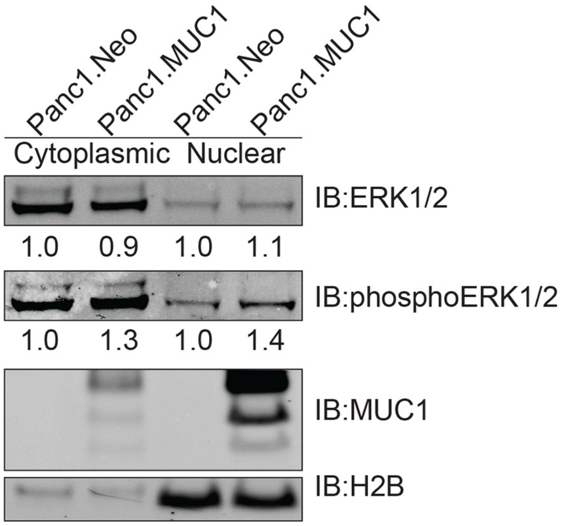

Supplementary Figure S1: Western blot analysis evaluating the expression of total ERK1/2 and phosphoERK1/2 in Panc1 cells. Relative amounts were determined by densitometry using H2B as a loading control. Calculations were performed for each localization and are for ERK2 only. Results show increased levels of phosphoERK with MUC1 expression.

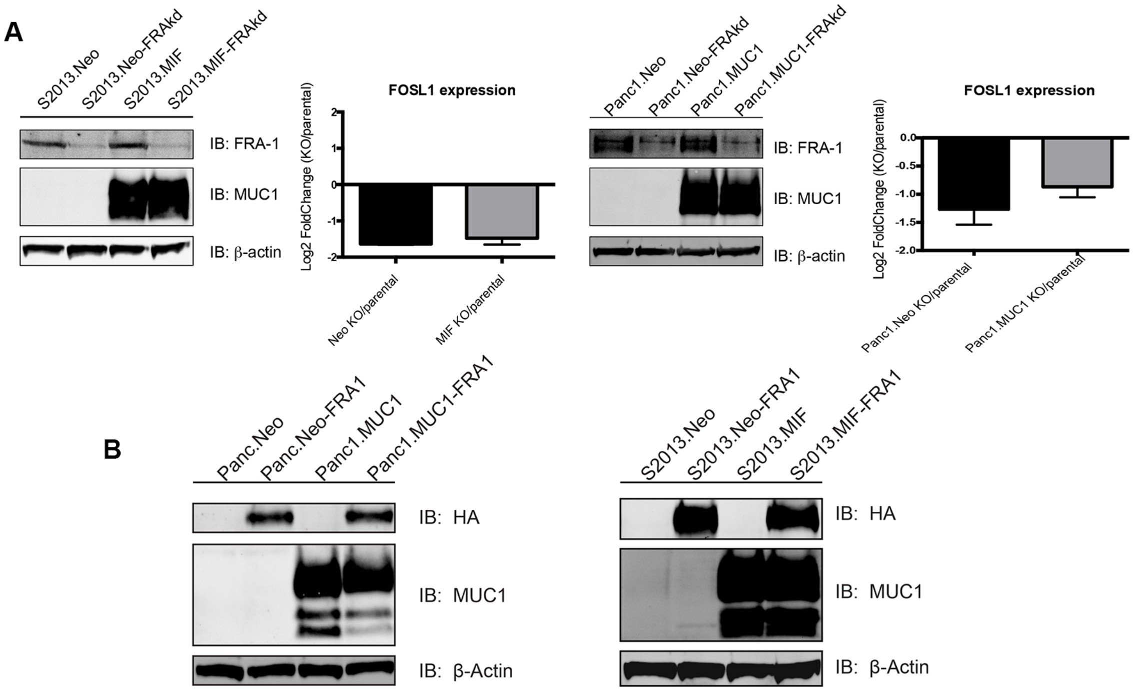

Supplementary Figure S2: (**A**) Characterization of FRA-1 knockdown cell lines. FRA-1 experession was knocked down by shRNA and expression of FRA-1 was assessed by both western blot analysis and RT-PCR. Reduction in FRA-1 expression was similar between the Neo and MUC1 expressing counterparts. (**B**) Characterization of FRA-1 levels following stable overexpression of HA-tagged FRA-1 construct. Lysates were probed for FRA-1 expression using an anti-HA antibody and MUC1. Actin served as a loading control. Both the Panc1 and S2013 cell lines express similar levels of FRA-1 independent of MUC1 expression allowing for comparisons across cell lines.

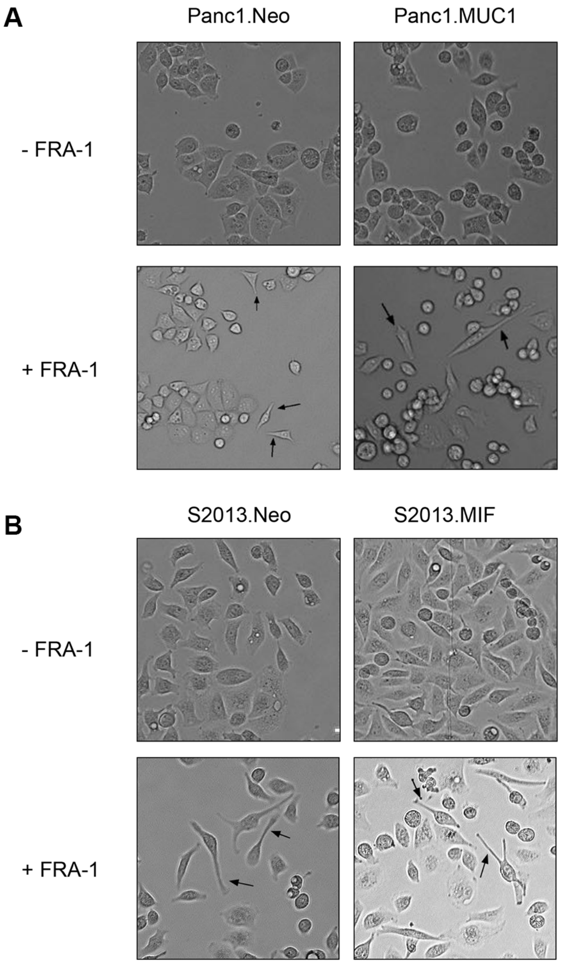

Supplementary Figure S3: Images of cancer cells with and without FRA-1 overexpression were taken using an EVOS digital microscope. Cells were than examined for changes in cellular morphology. Both Panc1 (**A**) and S2013 cells (**B**) exhibit increased numbers of cells with cellular projections when FRA-1 was overexpressed. (indicated by arrows).

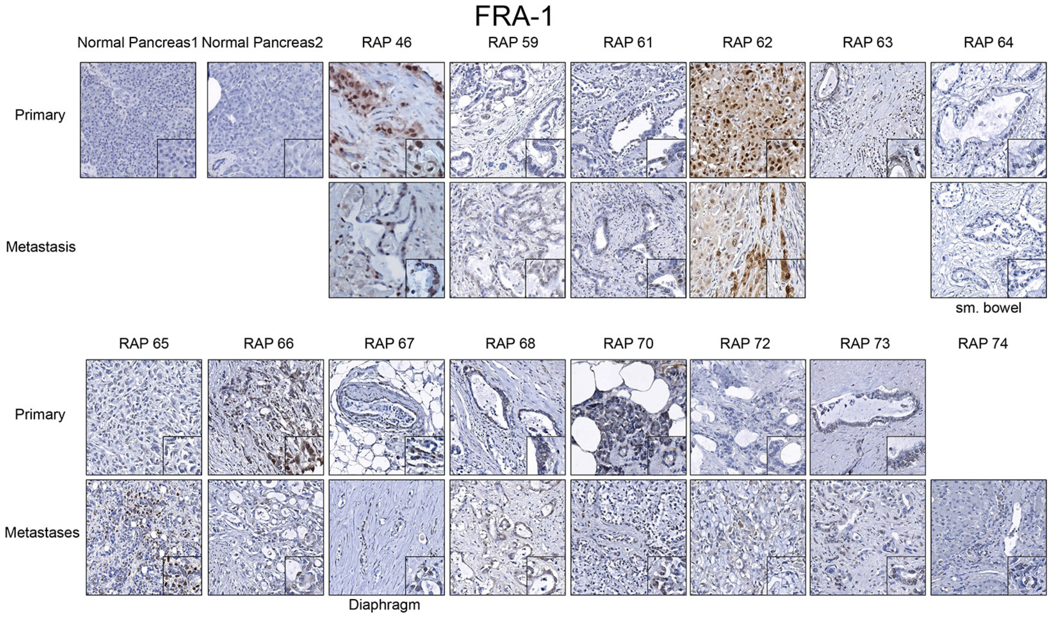

Supplementary Figure S4:Tissue microarrays were stained for expression of FRA-1. Samples were collected from the UNMC Rapid Autopsy Program. Each number represents an individual patient. Primary and metastatic sites were stained and compared to normal pancreas. Metastatic sites were collected from the liver unless otherwise indicated.

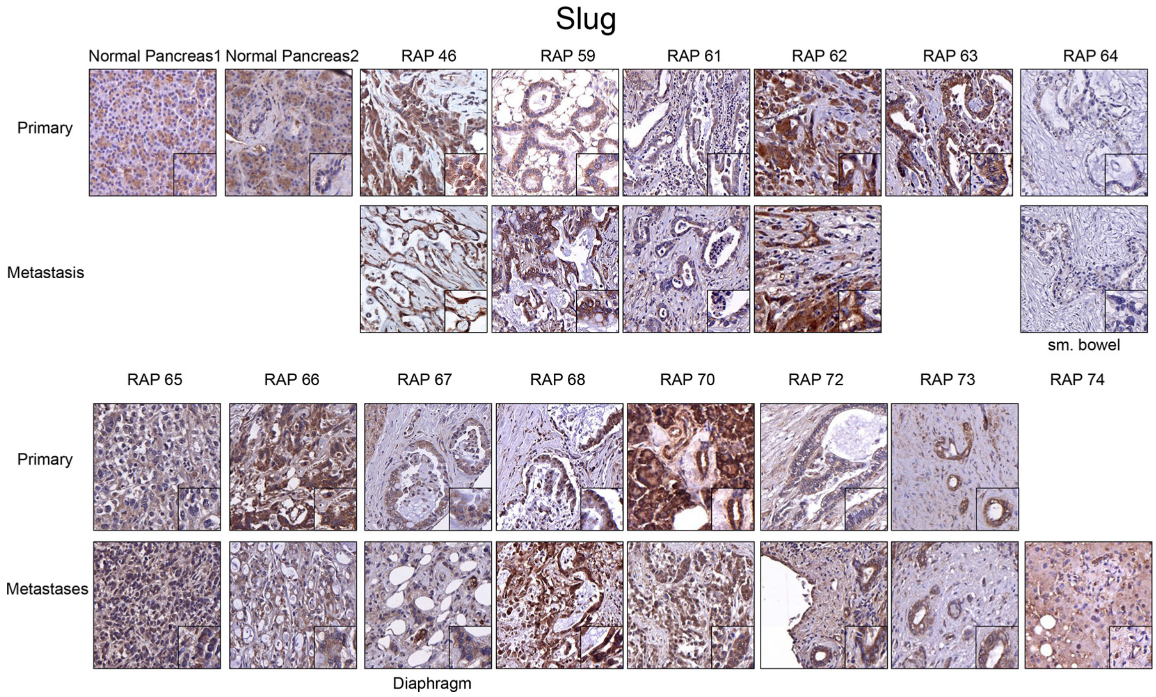

Supplementary Figure S5: Tissue microarrays were stained for expression of Slug. Samples were collected from the UNMC Rapid Autopsy Program. Each number represents an individual patient. Primary and metastatic sites were stained and compared to normal pancreas. Metastatic sites were collected from the liver unless otherwise indicated.

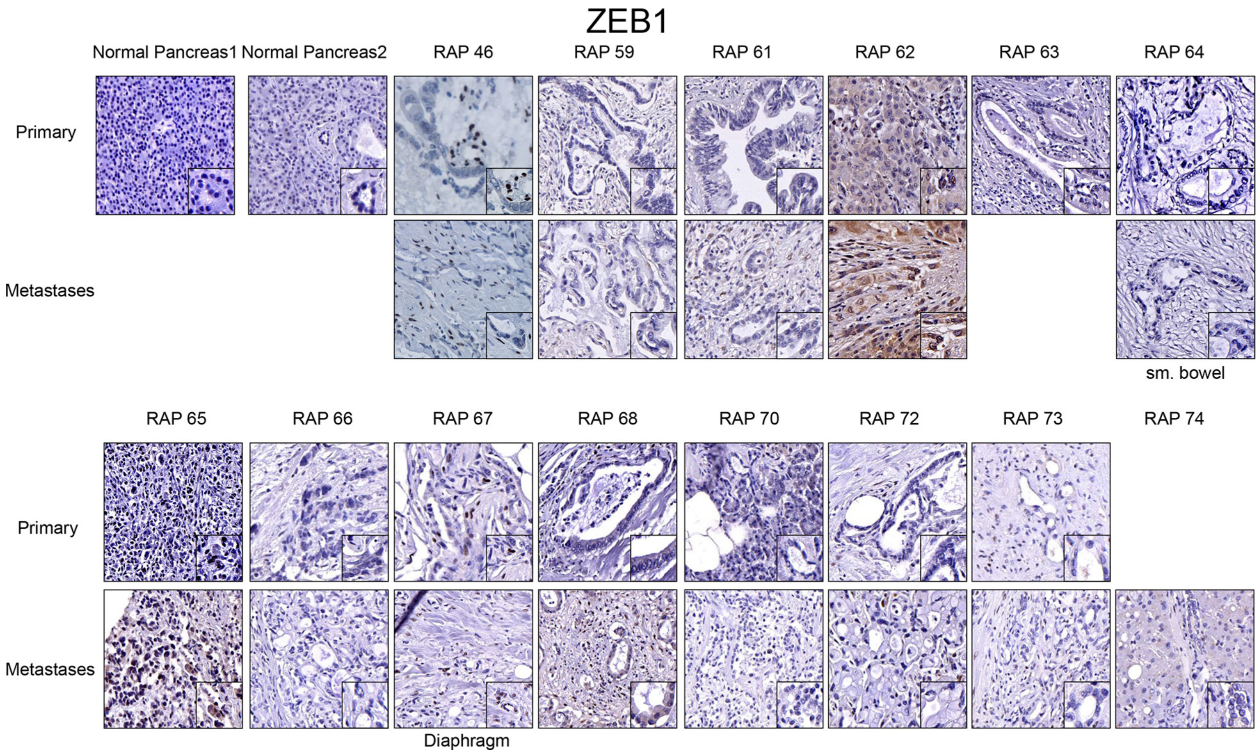

Supplementary Figure S6: Tissue microarrays were stained for expression of ZEB1. Samples were collected from the UNMC Rapid Autopsy Program. Each number represents an individual patient. Primary and metastatic sites were stained and compared to normal pancreas. Metastatic sites were collected from the liver unless otherwise indicated.

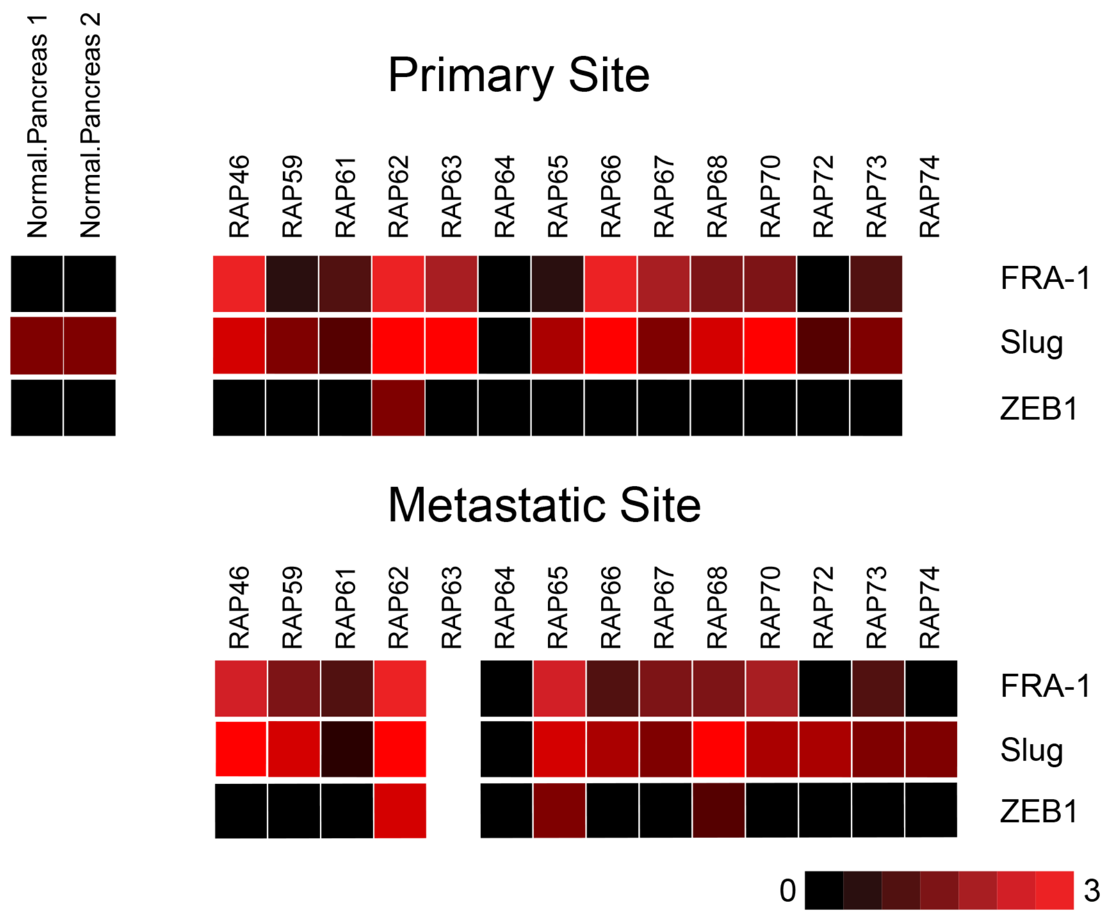

Supplementary Figure S7: Heatmaps were generated based upon staining of FRA-1, Slug, and ZEB1 in pancreatic cancer samples. Scoring was based upon relative intensity of staining and each TMA was scored independently to avoid bias based upon antibody differences. Scoring was restricted to tumor cells.
